# A New Method to Generate Artificial Frames Using the Empirical Mode Decomposition for an EEG-Based Motor Imagery BCI

**DOI:** 10.3389/fnins.2018.00308

**Published:** 2018-05-11

**Authors:** Josep Dinarès-Ferran, Rupert Ortner, Christoph Guger, Jordi Solé-Casals

**Affiliations:** ^1^Data and Signal Processing Research Group, Department of Engineering, University of Vic-Central University of Catalonia, Barcelona, Spain; ^2^g.tec Medical Engineering Spain SL, Barcelona, Spain; ^3^g.tec Medical Engineering GmbH, Schiedlberg, Austria

**Keywords:** brain-computer interface, motor imagery, empirical mode decomposition, artificial frames, EEG

## Abstract

EEG-based Brain-Computer Interfaces (BCIs) are becoming a new tool for neurorehabilitation. BCIs are used to help stroke patients to improve the functional capability of the impaired limbs, and to communicate and assess the level of consciousness in Disorder of Consciousness (DoC) patients. BCIs based on a motor imagery paradigm typically require a training period to adapt the system to each user's brain, and the BCI then creates and uses a classifier created with the acquired EEG. The quality of this classifier relies on amount of data used for training. More data can improve the classifier, but also increases the training time, which can be especially problematic for some patients. Training time might be reduced by creating new artificial frames by applying Empirical Mode Decomposition (EMD) on the EEG frames and mixing their Intrinsic Mode Function (IMFs). The purpose of this study is to explore the use of artificial EEG frames as replacements for some real ones by comparing classifiers trained with some artificial frames to classifiers trained with only real data. Results showed that, in some subjects, it is possible to replace up to 50% of frames with artificial data, which reduces training time from 720 to 360 s. In the remaining subjects, at least 12.5% of the real EEG frames could be replaced, reducing the training time by 90 s. Moreover, the method can be used to replace EEG frames that contain artifact, which reduces the impact of rejecting data with artifact. The method was also tested on an out of sample scenario with the best subjects from a public database, who yielded very good results using a frame collection with 87.5% artificial frames. These initial results with healthy users need to be further explored with patients' data, along with research into alternative IMF mixing strategies and using other BCI paradigms.

## Introduction

Brain-Computer Interfaces (BCI) are systems capable of controlling external devices using direct measures of the brain signals (Wolpaw et al., 2002; Wolpaw and Wolpaw, 2012). A BCI has three main parts:

1. Brain signals acquisition system.
2. Processing system.
3. Device/feedback control.

The selection of the brain signal acquisition system relies on the intended BCI application (Wolpaw et al., 2002; Shih et al., 2012; Wolpaw and Wolpaw, 2012). EEG is a non-invasive approach with a high temporal resolution that is suited for real-time application (Shih et al., 2012). EEG signals are electrical potential differences from different areas of the scalp caused by the firing of different neurons, often in response to an external stimulus. The resulting synchronized activity across large groups of neurons leads to electrical changes over different brain regions that can be recorded and sent to the processing system.

**Figure 1:**
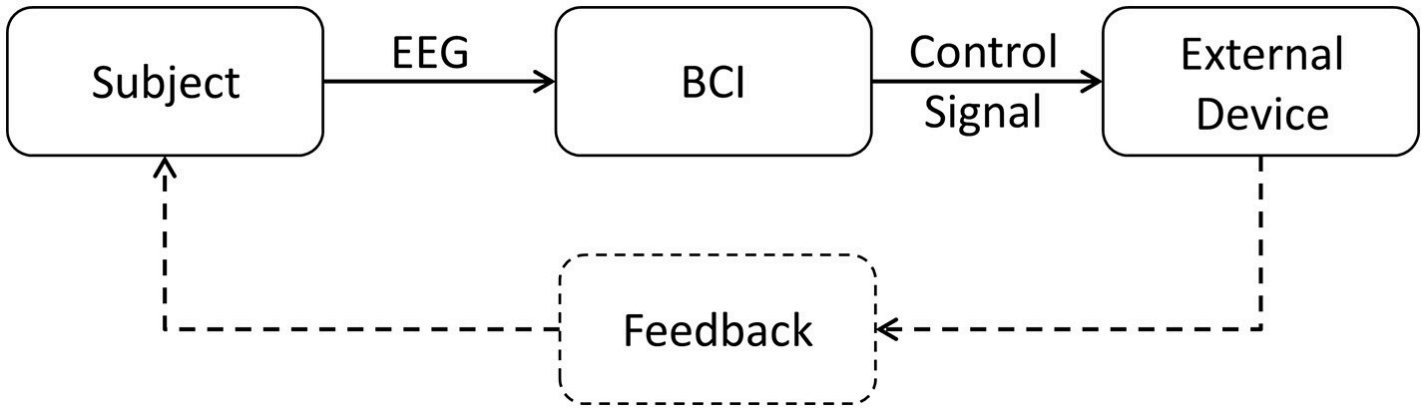
Block diagram of a generic EEG-based BCI system. The BCI gets EEG data from the subject, processes it and generates the proper signals to control the external device and give feedback to the subject.

In a BCI system (Figure 1), EEG signals are processed by a computer or processing unit (processing system). These signals are highly noisy, and the use of filtering and pattern recognition techniques are needed to acquire useful information from them (Wolpaw et al., 2002; Wolpaw and Wolpaw, 2012). Paradigms are instructions that the BCI user must follow to elicit known brain responses that the processing system can detect and use to control an external device. Many BCIs are designed to control monitors, but BCIs have been used with other external devices, such as functional electrical stimulator (FES) or orthosis as part of a BCI-based motor rehab system.

Recently, EEG-based BCIs have been extended to new tools for neurorehabilitation patients who have upper limb impairment due to a stroke (Ramos-Murguialday et al., 2013; Cho et al., 2016). They are also being used for patients with disorders of consciousness to assess their mental state and provide communication (Guger et al., 2013, 2017).

Different BCIs have used different paradigms (Farwell and Donchin, 1988; Pfurtscheller, 2001; Oehler et al., 2008), and one of the most widely used involves Motor Imagery or MI (Guger et al., 2015). In an MI BCI paradigm, the user is asked to imagine specific movements, such as left or right hand movements. This movement imagination activates areas of the motor cortex, much like the activation resulting from real movement. Thus, MI BCIs may determine whether a user is imaging left vs. right hand movement to provide a “yes” or “no” reply to a question or move a cursor horizontally.

In the MI paradigm, a trial is the time period which the user imagines movement, as well as any additional time needed for instructions, cues, or other delays. The BCI presents real-time feedback to the user that indicates how well the MI task is being performed and classified. This feedback might be visual information displayed on a screen, auditory feedback through headphones or proprioceptive or other feedback from other devices.

When using the MI BCI approach to help patients regain movement, the feedback often includes an avatar presented on a monitor that performs simulated hand/arm movements and FES electrodes placed over the affected limb. In conventional therapy, the patient is asked to imagine performing a movement such as wrist dorsiflexion while a therapist provides instructions and manages an FES device that triggers wrist dorsiflexion. By adding the MI BCI into the control loop, rewarding feedback such as avatar movement and FES activation is only possible when the patient performs the correct MI. This BCI-based feedback is much more tightly coupled to each patient's MI than conventional means, which should increase the functional improvement from therapy training (Remsik et al., 2016; Sabathiel et al., 2016).

BCIs, especially MI BCIs, usually require calibration for each user for at least two reasons. First, classifiers need time to learn the unique features of each new user's EEG activity, such as ERD/S used in MI BCIs. Second, these features may change within or across sessions or runs due to fatigue, medication, motivation, different cap placement, or other factors. Different cap placement from one session to another could be especially problematic if BCIs gain wider clinical adoption. Many therapists and other staff are not trained in precise cap positioning, and this process can require a few additional minutes. Calibration at the start of a session can lead to better classifier performance, but also requires additional time. Since MI BCIs typically require more calibration time than other BCIs, and patients with stroke may have limited time and motivation, new approaches to reduce calibration time with MI BCIs are needed.

In a typical BCI, a new EEG data frame is obtained from each trial. The quality of the classifier is directly proportional to the number of frames from each type of MI (such as left vs. right hand; Ramoser et al., 2000). This paper explores a new approach that creates artificial frames, which the classifier can use like real frames to reduce the need for calibration data. Because of the non-linear and non-stationary aspects of EEG signals, a new processing method based on the EMD decomposition (Huang et al., 1998) is proposed to generate those new artificial frames (Hawley et al., 2008; Huang et al., 2013; Riaz et al., 2015).

## Materials and methods

### Subjects

The experiment was performed on 7 healthy men aged 29.8 ± 5.76 years. All subjects reported no history of stroke or other cause of movement disability and signed an informed consent document prior to participating in the study.

### Equipment

The paradigm was implemented using a closed-loop system that provides real-time feedback to the user and saves the data for later analysis. This system uses a 16 EEG channel cap (g.SCARABEO, g.tec medical engineering GmbH) with the electrodes placed over the sensorimotor cortex according to the 10/10 international system: FC5, FC1, FCz, FC2, FC6, C5 C3, C1, Cz, C2, C4, C6, CP5, CP1, CP2, CP6. The Fpz electrode is connected to the ground and a reference electrode is placed on the right earlobe. The EEG cap is connected to a biomedical amplifier (g.USBamp, g.tec medical engineering GmbH), which is connected to a computer using a USB cable. The system provides two kinds of real-time feedback: a visual feedback through an avatar displayed on a screen, and proprioceptive feedback through FES electrodes placed on the extensor digitorum communis muscles of each subject's left and right arms.

### Experimental paradigm

At the beginning of each session, each subject was seated in a comfortable chair about 1 m in front of a monitor. The EEG cap was mounted and FES electrodes were affixed to both arms to stimulate wrist dorsiflexion. The experimenter visually inspected the subject's real-time EEG to check data quality and calibrated the FES parameters (pulse width and current) for each subject. Each subject was then asked to sit in front of the monitor and follow the instructions provided by the system.

**Figure 2:**
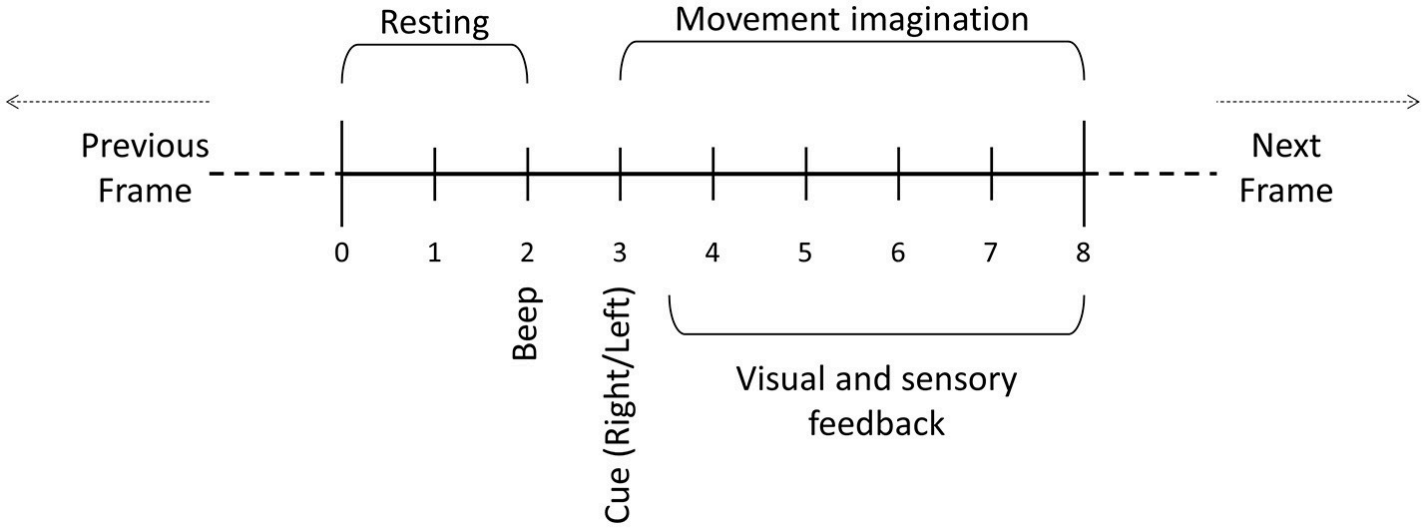
Motor imagery paradigm trial. During the first 2 s, the user is asked to relax. After 2 s, a beep is played and then an auditory cue indicates whether the user should imagine left or right movement. One frame consists of the data resulting from one trial.

Each subject completed one session with two runs. A short break was provided between these two runs, during which the subjects remained seated with the cap and FES electrodes in place. Each run presented 80 trials (40 for each side) to each subject. During the first 2 s of each trial, the subject rested, after which an acoustic signal (beep) indicated whether the subject should imagine left or right wrist dorsiflexion. The subject imagined the movement from seconds 3 to 8 while the system provided real-time feedback through the monitor and FES electrodes. After second 8, the trial ended and a new trial began (Figure 2). There were an equal number of cues to the left vs. right wrist during each run, and the order was chosen pseudorandomly. Data were stored for later offline analysis.

### Empirical mode decomposition

Common analytical tools like FFT and wavelets would not be adequate to process EEG signals in this scenario because they are non-linear and non-stationary. The Empirical Mode Decomposition (EMD) method is based on an algorithm that allows users to conduct a data-driven analysis that is more fitting with non-stationary signals that have changes in the frequency structure within a short period of time.

The algorithm decomposes the original signal into a finite number of functions called IMFs (Intrinsic Mode Function) that each of which represents a non-linear oscillation of the signal (Huang et al., 1998). Theses intrinsic functions fulfill two conditions:

1. In the whole signal, the number of maxima is the same as the number of zero-crossing, or differs by at most one.
2. For any sample, the mean value between the envelope of the local maxima and the envelope of the local minima is zero.

The process to obtain the IMFs from a signal *x*(*t*) is:

1. Set *s*(*t*) = *r*_*i*−1_(*t*). Initially, *i* = 1 and *r*_0_(*t*) = *x*(*t*).
2. Detect the local maxima and the local minima of *s*(*t*).
3. Interpolate all local maxima to generate the upper envelope.
4. Interpolate all local minima to generate the lower envelope.
5. Obtain the local mean *m*(*t*) by averaging the upper and lower envelopes.
6. Get a candidate for IMF by subtracting the local mean *m*(*t*) from the signal: *h*(*t*) = *s*(*t*) − *m*(*t*).
7. If *h*(*t*) does not satisfy the IMF's conditions, begin a new loop from step 2, setting *s*(*t*) = *h*(*t*).
8. Otherwise, *h*(*t*) is defined as an IMF: *IMF*_*i*_(*t*) = *h*(*t*).
9. *r*_*i*_(*t*) = *r*_*i*−1_(*t*) − *IMF*_*i*_(*t*).
10. If *r*_*i*_(*t*) is a monotonic function or does not have enough extrema to calculate the upper and lower envelopes, then *IMF*_*i*_(*t*) is the last IMF function of *x*(*t*) and the decomposition ends.
11. Otherwise, set *s*(*t*) = *r*_*i*_(*t*) and start a new loop from step 2 in order to obtain *IMF*_*i*+1_(*t*).

**Figure 3:**
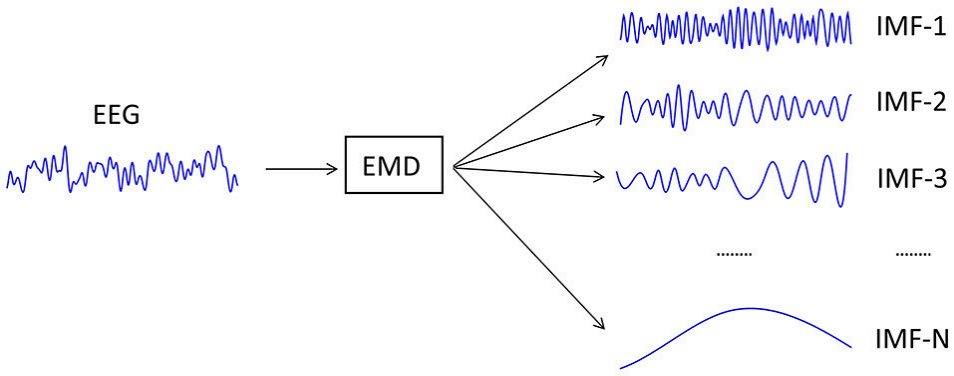
Decomposition of an EEG signal into all of its IMFs.

Once all the IMFs have been calculated, the signal can be recovered using its IMFs (1) and the final residue *r*_*n*_(*t*), where n is the number of extracted IMFs (Figure 3).

(1) $$\begin{array}{r} x\left(t\right)=\overset{n}{\underset{k\;=\;1}{\sum }}IMF_{k}\left(t\right)+r_{n}\left(t\right) \end{array}$$

The number of IMFs depends on the structure of the EEG signal, and may vary among different EEG data samples. An EEG signal is completely restored by adding all its IMFs and the final residue. Likewise, if a single one of these IMFs is replaced with another IMF from other previously decomposed EEG signal, using the formula (1), then a different EEG signal is obtained.

### New artificial EEG frames

Prior work has created EEG artificial frames using some stationary approaches that use Gaussian noise as a source into an FFT-based system (Paris et al., 2017), but this approach lacks the temporal features of the natural EEG signals. Otherwise, in some studies the artificial EEG is created by mixing different parts of different temporal EEG signals (Lotte, 2011). In this case, the method keeps the temporal features of the signal, but without control of its frequency features.

Using the EMD approach, the new artificial EEG signals can be created by combining some IMFs from different real EEG signals. Although those new EEG signals will be different from the real ones, they will exhibit similar features and the same underlying structure. Unlike the other approaches described above, the EMD analysis can keep the features within temporal and frequency domains, because each IMF is a representation in the temporal domain of a specific non-linear oscillation of the signal.

In the paradigm used in this study, each MI frame is composed of 16 EEG signals, meaning that any new artificial frame needs 16 new artificial EEG signals.

**Figure 4:**
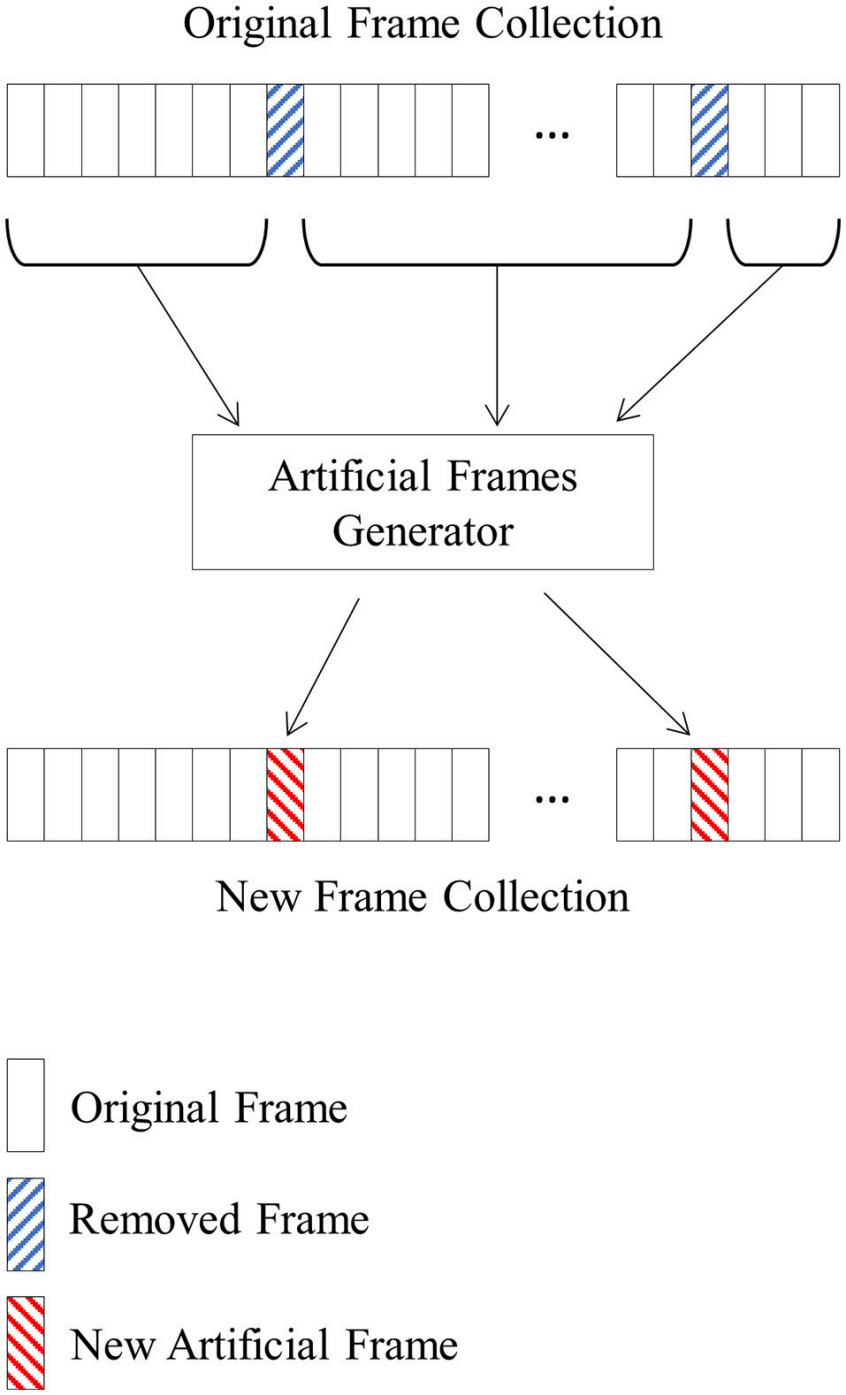
A new frame collection containing artificial frames is created using an original frame collection and randomly selecting the removed frames. The IMFs of the non-selected frames are randomly mixed to create the artificial frames that will replace the removed ones.

Starting from a real frame collection, the new frame collection containing artificial frames is built following these steps:

1. Define the number of frames to be replaced. This requires replacing the same number of frames from each class (right-side and left-side) with a maximum of 40 frames.
2. Randomly select the frames to be replaced in the original frame collection. The rest of the frames contribute with their IMFs to build the new artificial frames.
3. The selected frames are split in two sets of frames according to their class (left vs. right).
4. To create an artificial frame of a specific class, a number of N frames are selected randomly from the set of frames belonging to the same class (Figure 4). The first selected frame contributes with all its first IMFs (16 IMFs, one per channel), the second one with its second IMFs, and successively until the nth frame, which contributes with its nth IMFs.
5. Add up all the IMFs corresponding to the same channel to build each new EEG channel of the new artificial frame.

Repeat step 4 for each new artificial frame necessary to complete the frame collection.

As explained in section Empirical Mode Decomposition, different EEG signals might have different numbers of IMFs, and it is necessary to establish beforehand the number of IMFs of the new artificial frames. In this study, we considered that an EEG signal to be completely represented using their first 15 IMFs, because none of the decomposed signals had more than 12 IMFs. Thus, in every real decomposed EEG signal with <15 IMFs, additional zero value IMFs were added, reaching 15 IMFs for every decomposed signal.

We used this procedure to create new frame collections for each subject's data. Each of these new frame collections contained a different number of artificial frames: 2 (2.5%), 4 (5%), 6 (7.5%), 8 (10%), 10 (12.5%), 20 (25%), 30 (37.5%), or 40 (50%). This process created 9 frame collections: the original data with 0 artificial frames, and eight collections with artificial frames. For each of those 9 frame collections, we constructed a classifier and determined the error rate.

### Classifier training and implementation

The classifier is based on Linear Discriminant Analysis (LDA). Initially, the frame collection is divided in two groups of frames according to their class (right or left wrist movement). Next, every signal is bandpass filtered (8–30 Hz) and then artifact rejection is applied. With the non-rejected frames, a spatial CSP filter is calculated (Koles et al., 1990; Wang et al., 2005), keeping only the 2 first and 2 last result vectors as the spatial filter. Therefore, the 16 EEG signals of a frame are spatially filtered resulting in four signals. A 1.5 s window variance is calculated over each of these signals. Finally, these variances are normalized and scaled logarithmically, then used as features to build the LDA classifier (Cho et al., 2016).

A frame collection and classifier are needed to calculate the error rate. Each frame is passed through the classifier, which outputs a value indicating the estimation of that frame's class for each one of its 2,048 samples (256 samples a second). This result is then compared to the true class and marked as correct if they match, and incorrect otherwise. After determining the error of every single sample of a frame collection, a percentage of the incorrect samples is calculated over the feedback period of each trial (from second 3.5 to second 8), providing the global error rate for that classifier. The error rate is expressed as two different percentage values: right-side error rate and left-side error rate.

**Figure 5:**
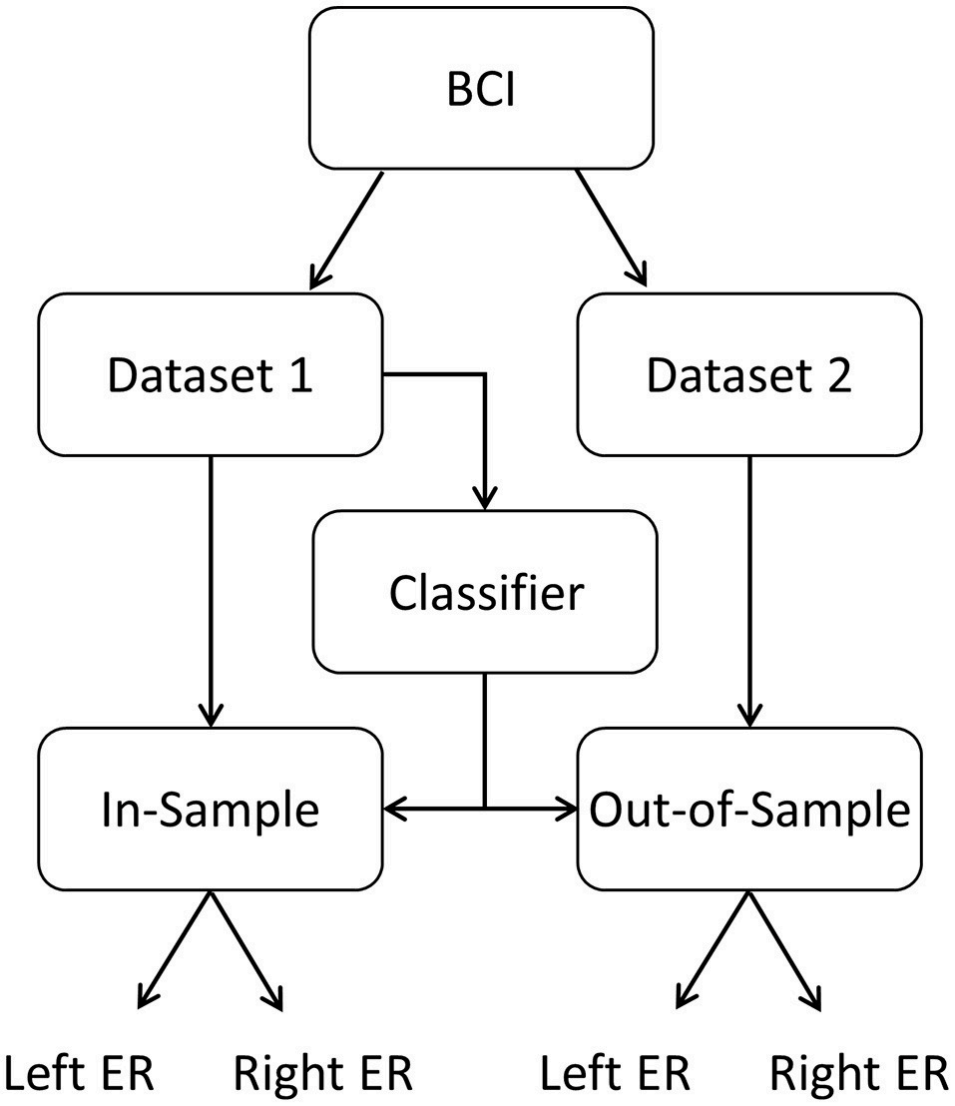
The paradigm provided two datasets. The first dataset was used to build the classifier. Next, the classifier was assessed with both datasets: in-sample (dataset 1) and out-of-sample (dataset 2). Left-side and right-side error rate (ER) can then be determined to assess classifier performance.

Data from each subject's first run were used to build all the classifiers, and data from the second run were used to assess the performance of these classifiers with out-of-sample data (Figure 5). The out-of-sample error rate of the classifiers without artificial frames were also calculated.

The new frame creation process relies on the random selection of the removed frames and the IMFs. Repeating the experiment with a different random seed leads to different frame collections and very likely to slightly different results. Hence, the frame creation procedure in section New Artificial EEG Frames and classification process described in this section were repeated 100 times for each subject.

### Median absolute deviation

The MAD (Median Absolute Deviation) is a method to detect outliers from a statistical sample when the sample is small and has a non-normal distribution (Leys et al., 2013); instead of using the mean values to fix the boundaries it uses the median value. Usually, the upper boundary is defined as three times the MAD above the median, and the lower one as three times below (2). All samples outside those boundaries are considered as outliers, and all inside ones as inliers (3).

(2) $$\begin{array}{r} M-3\times MAD\;<x<M+3\times MAD \end{array}$$

(3) $$\begin{array}{r} |\frac{x-M}{MAD}|<3 \end{array}$$

We used the MAD approach to validate the performance of each classifier with a specific number of artificial frames. We used the MAD and the sample's median to calculate a ratio (4), and two values of this ratio were obtained using the error rates of the classifiers built without artificial frames.

(4) $$\begin{array}{r} R=|\frac{x-M}{MAD}| \end{array}$$

For example, after 100 repetitions of the experiment for a specific subject, 100 classifiers with N artificial frames were created (using a frame collection with N artificial frames), and their right and left error rates were calculated. From this sample, the median and the MAD values were obtained. Then, the ratio R was calculated using the error rates of the classifier created with the frame collection without artificial frames.

This process sought to determine whether the original classifier could be considered as an inlier of the sample of the classifiers with N artificial frames. Thus, values of R below 3 meant that the original classifier was not an outlier and the replacement of the real frame collection with artificial ones is similar for this specific subject and with a maximum of N artificial frames.

## Results

### In sample results

A classifier with a specific number of artificial frames is considered similar to its original if its right and left ratios are both below 3 (section Classifier Training and Implementation). Across all subjects and all classifiers, only one of the classifiers with 37.5% of artificial frames of subject S02 is considered as dissimilar (Table 1). From the same subject, the classifiers with 25.0 and 50.0% are just below 3. Using lower maximum ratios applied stricter conditions to test the classifiers. If we apply a ratio threshold of 2.6 instead of 3, these two outcomes from S02 would be considered an outlier. Further, subjects S03 and S06 also have high ratio values (above 2.6), but below 3. If a maximum ratio of 2 is applied, all the classifiers for all subjects were acceptable if the frames collection used at most 12.5% of artificial frames. All classifiers were statistically similar to their corresponding original classifiers for subjects S01, S04, S05, and S07.

**Table 1:** Ratio between the error rate for each side and its MAD (Median Absolute Deviation).

	**S01**		**S02**		**S03**		**S04**		**S05**		**S06**		**S07**	
**AF^a^**	**R^b^**	**L^c^**	**R**	**L**	**R**	**L**	**R**	**L**	**R**	**L**	**R**	**L**	**R**	**L**
2.5	0.12	0.67	0.22	0.64	0.58	1.27	0.32	0.31	0.32	0.27	0.33	0.64	0.34	0.69
5.0	0.05	1.03	0.82	0.56	1.11	1.02	0.46	0.45	0.18	0.35	0.47	0.83	0.01	0.63
7.5	0.29	0.88	1.03	0.07	1.06	1.51	0.51	0.51	0.00	0.02	1.17	1.49	0.46	0.62
10.0	0.37	1.13	0.99	0.11	1.19	1.75	0.80	0.46	0.38	0.08	1.04	1.66	0.49	0.84
12.5	0.24	0.94	1.42	0.04	1.89	1.86	1.00	0.44	0.46	0.27	0.87	1.52	0.40	0.85
25.0	0.09	1.44	2.79	0.44	2.13	1.94	1.28	0.61	0.96	0.78	0.71	2.09	0.51	1.28
37.5	0.11	1.55	3.12	0.41	1.97	2.01	1.20	0.69	1.07	1.18	0.57	2.66	0.73	1.92
50.0	0.15	1.45	2.86	1.00	2.18	2.68	1.27	1.06	1.42	1.23	0.62	2.76	0.73	1.86

a *AF, % of artificial frames in the classifier*.

b *R, right-side ratio*.

c *L, left-side ratio*.

Classifiers with more than 37.5% of artificial frames for subjects S01 and S06 showed a smaller ratio in the right-side class than the classifiers with fewer artificial frames. However, the left-side class of the same classifiers increased considerably.

### Out of sample results

The previously created classifiers and the second recorded dataset were used to analyze performance with out-of-sample data. First, we calculated the error rate of the classifiers built without artificial frames. We only designated the classifiers with an error rate below of 33% in both sides as useful. Under these conditions, only subject S01 and S03 had valid error rates in both sides (Table 2).

**Table 2:** Error rate of the classifier built with the frame collection without artificial frames.

**S01**		**S02**		**S03**		**S04**		**S05**		**S06**		**S07**	
**R^a^**	**L^b^**	**R**	**L**	**R**	**L**	**R**	**L**	**R**	**L**	**R**	**L**	**R**	**L**
5.50	6.68	11.20	66.67	29.83	20.39	42.67	32.96	36.24	35.79	27.27	39.60	58.34	22.74

a *R, right-side error rate*.

b *L, left-side error rate*.

Table 3 presents additional details from subjects S01 and S03. Subject S01 showed very good results, with very small and similar error rates between the original classifiers and the rest of his classifiers. Subject S03 showed higher error rates than subject S01, and the error rates increased slightly with the number of the artificial frames in the frame collection (Table 3). Nonetheless, the classifiers built with at most 37.5% of artificial frames had error rates in both sides below the 33% threshold. However, the right-side error rate of classifier with 50% of artificial frames is 34.06%, meaning that this classifier should not be considered as valid.

**Table 3:** Error rate of classifiers built with frame collections with artificial frames.

	**S01**		**S03**	
**AF^a^**	**R^b^**	**L^c^**	**R**	**L**
0.0	5.50	6.68	29.83	20.39
2.5	4.02	7.52	22.20	18.64
5.0	4.06	7.46	22.21	19.20
7.5	3.80	7.66	21.94	20.79
10.0	3.79	7.63	24.52	21.52
12.5	3.76	7.77	24.92	19.96
25.0	3.47	8.05	28.15	24.75
37.5	3.86	8.70	31.59	25.79
50.0	3.79	8.84	34.06	31.39

a *AF, % of artificial frames in the classifier*.

b *R, right-side error rate*.

c *L, left-side error rate*.

Considering that only 2 out of 7 subjects were considered valid to be analyzed in an out of sample scenario, and that an error rate below 33% can still lead to a valid classifier, we also used an external EEG MI dataset (Cho et al., 2017) to increase the number of subjects. We selected the four subjects with best accuracies and split their dataset in two different sets of data. The first dataset was used to create the classifier, and the second dataset was used to calculate the out of sample error rate. Table 4 show the experimental results, which are very close to the results from the subjects recorded in the present study. Results are especially good for subjects E01 and E02. Subject E03 (only) showed a non-valid value in the classifier built with a density of 50%, meaning that all his other classifiers should be considered useful. On the other hand, subject E04 has no value below 33% and any classifier should be considered valid.

**Table 4:** External datasets.

	**E01**		**E02**		**E03**		**E04**	
**AF^a^**	**R^b^**	**L^c^**	**R**	**L**	**R**	**L**	**R**	**L**
0.0	12.82	11.43	2.99	18.08	14.56	21.08	4.65	31.51
2.5	12.68	10.85	3.12	17.87	14.04	21.50	4.04	33.49
5.0	12.88	10.65	2.98	18.30	15.37	22.93	4.34	33.66
7.5	13.21	10.48	3.36	17.90	15.13	21.83	4.42	36.77
10.0	13.36	10.80	3.50	17.36	14.65	22.69	4.91	35.91
12.5	13.19	10.64	3.47	17.70	15.54	24.98	4.85	38.27
25.0	14.74	11.10	3.66	17.00	19.47	27.18	7.11	37.38
37.5	15.45	11.72	4.73	16.40	20.90	32.44	7.46	39.52
50.0	15.80	13.34	6.24	17.06	31.04	34.32	9.87	38.20

*Out of sample error rates of classifiers built with artificial frames*.

a *AF, % of artificial frames in the classifier*.

b *R, right-side error rate*.

c *L, left-side error rate*.

### Additional out of sample results

In the previous experiments we used a maximum density of artificial frames of 50%. Here we present new experiments increasing this density above 50% in order to determine the subject-specific maximum density possible that can still yield valid classifiers (both mean error rates below 33%). The experiment was repeated for densities of 62.5, 75, and 87.5%. As shown in Table 5, subjects S01, E01, and E02 had error rates below 33% with a frame collection composed of 87.5% of artificial frames and below. Subject E04 has no valid classifier, and the other two subjects (S03 and E03) showed error rates above 33% with densities above 50%. However, data from subject E04 had not yielded any valid classifier in the latter results with densities up to 50%.

**Table 5:** Additional results.

	**S01**		**S03**		**E01**		**E02**		**E03**		**E04**	
**AF^a^**	**R^b^**	**L^c^**	**R**	**L**	**R**	**L**	**R**	**L**	**R**	**L**	**R**	**L**
0.0	5.50	6.68	29.83	20.39	12.82	11.43	2.99	18.08	14.56	21.08	4.65	31.51
25.0	3.47	8.05	28.15	24.75	14.74	11.10	3.66	17.00	19.47	27.18	7.11	37.38
50.0	3.79	8.84	34.06	31.39	15.80	13.34	6.24	17.06	31.04	34.32	9.87	38.20
67.5	10.11	10.76	36.04	46.75	16.15	16.63	7.30	19.28	31.85	41.76	13.15	39.34
75.0	17.98	12.86	39.07	47.67	19.05	18.93	10.87	20.54	34.32	47.03	15.67	44.33
87.5	17.67	28.27	45.30	45.36	23.54	32.25	17.25	26.99	36.56	51.06	26.26	46.39

*Out of sample error rate of classifiers built with artificial frames*.

a *AF, % of artificial frames in the classifier*.

b *R, right-side error rate*.

c *L, left-side error rate*.

## Discussion

This paper introduced a new method to create EEG artificial data frames to reduce the calibration time required for a MI BCI paradigm. The results suggest that the maximum number of artificial frames that are advisable in a frame collection varies substantially across different people. This could occur because the subject's MI varies within and across each trial, meaning that the mixing of different IMFs might produce a less helpful artificial frame. Longer training should help subjects learn to generate more consistent and distinct MI activity, and shorter trials and improved feedback could also be helpful.

The in-sample results demonstrate that the method is useful when creating similar classifiers for four out of seven subjects when the frame collection has at most 50% of artificial frames, which allows halving the training time for these subjects. This could reduce fatigue, stress and discouragement associated with the training, when feedback is often inaccurate. Additional research might identify methods to identify priori which subjects could tolerate frame collections with 50% or even more artificial frames.

While in-sample results are used to assess the capability of the neurorehabilitation patient or other users to control the BCI, out-of-sample processing is used to send the feedback to the patient. Typically, the BCI uses a classifier created from the preceding session from the patient. Reducing the error rate in out-of-sample data results in more accurate feedback, which should improve the closed-loop synergy between the user and the BCI. Out-of-sample results showed that subjects whose classifiers based on real data yielded acceptable error rates (below 33%) also had acceptable error rates when using the classifiers with artificial frames. However, only 2 out of the 7 subjects had original classifier error rates below 33%, which is insufficient to thoroughly validate this method on an out-of-sample environment.

Our study also included four subjects with good MI accuracy from an external database. Their out-of-sample error rates were very close to the ones achieved with the subjects of our study. Seeing these good out-of-sample results, we extend the experiment with densities beyond 50%. In 3 of these 6 subjects, the results showed that classifiers built with 87.5% of artificial frames still led to error rates below 33%. Additional research will be needed to explore whether the slight increase in error rate resulting from the increase of artificial frames in the frame collection is worth the reduced training time. Further research could also enlarge the density of artificial frames, which may help increase the generalization of the classifiers and thereby decrease their out of sample error rates.

The study showed a similar in-sample behavior in all subjects' classifiers created with a maximum of 12.5% of artificial frames in their frame collections and a strict ratio threshold of 2. Using 12.5% artificial frames would improve a motor imagery BCI system in two ways. First, it would reduce the training time from 720 to 630 s. Second, the method could be used to replace an artifacted frames with artificial ones. In the CSP calculation, the number of frames for each side must be exactly the same, and if there are some artifacted frames in one class, the number of frames in the other class must be reduced accordingly. This can reduce classifier accuracy and may necessitate additional training runs. Instead, up to 12.5% of artifacted frames could simply be replaced.

This study used an LDA classifier due to its widespread use in MI BCI paradigms. Further studies could explore test the artificial frame creation method using different classifiers. Another interesting direction is the mixing strategy of the IMF to obtain the artificial frames. The described method mixes 15 IMF from different 15 randomly chosen real frame to build a new artificial frame. Mixing only the most significant IMFs (instead of fifteen), or even reducing the number of real frames to three or four, might both be worth exploring.

This approach might also be extended to other types of BCIs. For example, some passive approaches for evaluating alertness or fatigue might benefit. BCIs based on the P300 complex, steady-state evoked potentials, and similar BCI paradigms that require focused attention typically require much less training than MI and most other BCIs. However, this approach could still be useful for countering artifact or to improve classifier accuracy in some users, such as patients using a vibrotactile P300 system.

Most importantly, this new BCI method needs additional research with more subjects, especially to validate the out-of-sample behavior. These subjects should include target patients, including persons with stroke and other persons seeking rehabilitation. New paradigms could provide training of other types of rehabilitation, such as lower-limb training. Patients with locked-in syndrome (LIS) may also benefit from this approach for communication or other goals.

## Ethics statement

This study was carried out in accordance with the recommendations of the Ethics Committee of the Kepler Universitätsklinikum (Kepler University Hospital), Austria. The protocol was approved by the Ethikkommission des Landes OberÖsterreich (Ethics Committee of the Province of Upper Austria). All subjects gave written informed consent in accordance with the Declaration of Helsinki.

## Author contributions

JS-C conceived and organized the artificial frame generation protocol and its application; JD-F collected the experimental data, implemented the protocol and the classification algorithm, and performed the statistical data analysis; RO contributed to the signal processing section; JS-C and CG had theoretical contributions on the analysis of the results; JD-F wrote the first draft of the paper. All authors reviewed the draft of the paper and approved the final manuscript.

### Conflict of interest statement

CG is the co-CEO of g.tec Medical Engineering. JD-F and RO are all employed by g.tec Medical Engineering. JS-C declares that the research was conducted in the absence of any commercial or financial relationships that could be construed as a potential conflict of interest.
